# Folate and Vitamin B_12_ Deficiency Exacerbate Inflammation during *Mycobacterium avium paratuberculosis* (MAP) Infection

**DOI:** 10.3390/nu15020261

**Published:** 2023-01-04

**Authors:** Joseph A. Vaccaro, Ahmad Qasem, Saleh A. Naser

**Affiliations:** Division of Molecular Microbiology, Burnett School of Biomedical Sciences, College of Medicine, University of Central Florida, 4110 Libra Drive, Orlando, FL 32816, USA

**Keywords:** Crohn’s disease, MAP, folate, cobalamin, B_9_, B_12_, paratuberculosis

## Abstract

Folate and vitamin B_12_ deficiency is highly prevalent among Crohn’s disease (CD) patients. Furthermore, CD pathology can be mediated by *Mycobacterium avium* subsp. *paratuberculosis* (MAP) infection. However, the direct effect of folate (B_9_) and cobalamin (B_12_) deficiency during MAP infection remains uncharacterized. This study investigates how folate and B_12_ deficiency impedes macrophage apoptosis and exacerbates the inflammation in macrophages infected with MAP isolated from CD patients. Accordingly, we measured folate and B_12_ in ex vivo plasma samples collected from CD patients with or without MAP infection (*N* = 35 per group). We also measured the expression of the pro-inflammatory cytokines IL-1β and TNF-α, cellular apoptosis and viability markers, and bacterial viability in MAP-infected macrophages cultured in folate and B_12_ deficient media. We determined that MAP-positive CD patients have significantly lower plasma folate and B_12_ in comparison to MAP-negative CD patients [414.48 ± 94.60 pg/mL vs. 512.86 ± 129.12 pg/mL, respectively]. We further show that pro-inflammatory cytokines IL-1β and TNF-α are significantly upregulated during folate and vitamin B_12_ deprivation following MAP infection by several folds, while supplementation significantly reduces their expression by several folds. Additionally, depletion of folate, B_12_, and folate/B_12_ following MAP infection, led to decreased macrophage apoptosis from 1.83 ± 0.40-fold to 1.04 ± 0.08, 0.64 ± 0.12, and 0.45 ± 0.07 in folate-low, B_12_-low, and folate/B_12_-low cells, respectively. By contrast, folate and folate/B_12_ supplementation resulted in 3.38 ± 0.70 and 2.58 ± 0.14-fold increases in infected macrophages. Interestingly, changes in overall macrophage viability were only observed in folate-high, folate/B_12_-high, and folate/B_12_-low media, with 0.80 ± 0.05, 0.82 ± 0.02, and 0.91 ± 0.04-fold changes, respectively. Incubation of Caco-2 intestinal epithelial monolayers with supernatant from infected macrophages revealed that folate/B_12_ deficiency led to increased LDH release independent of oxidative stress. Overall, our results indicate that folate and B_12_ are key vitamins affecting cell survival and inflammation during MAP infection.

## 1. Introduction

Folate (vitamin B_9_) and cobalamin (vitamin B_12_) are essential cofactors in eukaryotic single-carbon metabolism [1,2]. Folate is naturally found in citrus fruits and leafy green vegetables; it is also supplemented in particular grains and synthesized by intestinal commensal bacteria such as *Bifidobacterium* strains [3,4,5]. In contrast, vitamin B_12_ is primarily found in animal products [6,7]. These two cofactors serve distinct roles in normal cellular metabolism, since folate transfer of methyl groups is necessary for the biosynthesis of purines, formyltransferase reactions, and the conversion of uridine to thymidylate [8,9,10]. Similarly, vitamin B_12_ is a cofactor for converting methylmalonyl-CoA to succinyl-CoA, thus proving a necessity for odd-chain fatty acid and amino acid metabolism [2]. Furthermore, the central cobalt moiety in the vitamin B_12_ corrin ring scavenges cyanide ions and reactive oxygen species (ROS) [11,12,13,14]. Folate and vitamin B_12_ overlap in function during the regeneration of methionine from homocysteine in a reaction catalyzed by methionine synthase [15]. During this reaction, L-methyltetrahydrofolate donates a methyl group to homocysteine using B_12_ as a cofactor [15]. The methionine generated this way can subsequently be incorporated into protein or adenosylated to generate S-adenosyl methionine (SAM), which is the universal methyl donor and precursor to polyamine biosynthesis [16,17].

Several studies have shown that folate and B_12_ deficiencies are associated with exacerbated inflammation and damage in the brain, vasculature, immune system, liver, and gastrointestinal tract [18,19,20,21,22,23,24,25,26,27]. This phenomenon is partly due to their metabolic roles in regenerating methionine from homocysteine; homocysteine mediates inflammation via cathepsin V activation and TXNIP-induced NLRP3 inflammasome activation [28,29]. However, macrophages supplemented with folate in excess of standard cell culture concentrations display reduced pro-inflammatory gene expression [30]. These effects are also observed in vivo during macrophage-mediated neuroinflammation [31]. Therefore, folate and vitamin B_12_ may link hypovitaminosis and chronic inflammatory disease, particularly when the disease progression impedes vitamin uptake [21,32].

Crohn’s disease (CD) is a chronic inflammatory bowel disease characterized by a pattern of relapse and remission and asymmetrical, segmental, and transmural inflammation [33]. CD patients are at elevated risk of malabsorptive vitamin deficiency due to chronic damage to the gastrointestinal wall [21,32,34,35]. Recently, a murine model of CD has indicated that a diet enriched in folate and cobalamin enhances the antibacterial response to pathogenic *E. coli* infection [36]. However, the clinical effect of folate and vitamin B_12_ supplementation on pathogens associated with CD needs further investigation.

*Mycobacterium avium* subspecies *paratuberculosis* (MAP) is an obligate intracellular pathogen that is known as a causative agent in Johne’s disease (JD), which is a chronic inflammatory disorder affecting the intestines of ruminants [37]. In genetically susceptible patients, MAP infection induces CD, leading to chronic gastrointestinal inflammation that requires the use of immunosuppressive agents to manage disease symptoms [38,39,40]. Since standard CD therapy involves anti-TNF-α biologic drugs that fail to alleviate symptoms in roughly half of CD patients and exacerbate MAP infection in tissue culture, exploring alternative immunomodulatory interventions for this patient subset is an ongoing priority [41,42]. In this context, our present study analyzes the effects of folate and B_12_ supplementation or deprivation on MAP infection and the resultant inflammatory response.

## 2. Materials and Methods

### 2.1. Measurement of Plasma Folate and Vitamin B_12_

Plasma samples from peripheral blood (4.0 mL K_2_-EDTA tube) were collected from 100 CD patients (CDAI ≥ 220 and ≤450). The presence of MAP was subsequently evaluated via *IS900* PCR as described earlier [43]. We randomly selected 35 MAP-positive and 35 MAP-negative CD patients for this study. Within the selected samples, we used the Human Folic Acid ELISA Kit (Competitive EIA) (LifeSpan BioSciences, Seattle, WA, USA), the 5-Methyltetrahydrofolate ELISA Kit (LifeSpan BioSciences, Seattle, WA, USA), and the Human vitamin B_12_ (VB_12_) ELISA Kit (Aviva Systems Biology, San Diego, CA, USA) to determine levels of folate and vitamin B_12_, respectively. This study was approved by the University of Central Florida Institutional Review Board # STUDY00003468. All samples were de-identified before handling.

### 2.2. Culture and Infection of Monocyte-Derived Macrophages

THP-1 immortalized monocyte-like cells (ATCC TIB-202) were cultured in custom formulated folate and B_12_-free RPMI-1640 medium (ThermoFisher, Waltham, MA, USA) with 10% fetal bovine serum (FBS; Sigma Life Science, St. Louis, MO, USA). Pure folic acid and 1 mg/mL vitamin B_12_ solution in methanol (Sigma Life Science, St. Louis, MO) were then added to the medium to generate media with differential B-vitamin status (Table 1). The cells were grown to confluency in treated cell culture flasks in a humidified 5% CO_2_ incubator at 37 °C. A total of 2.0 mL of cell suspension was transferred to 12-well tissue culture plates with 1 × 10^5^ cells per well. We then differentiated the cells into monocyte-derived macrophages using 50 ng/mL phorbol 12-myristate 13-acetate (PMA; Sigma Life Science, St. Louis, MO, USA), followed by 48 h of incubation at 37 °C. In MAP-positive treatment groups, monocyte-derived macrophages were infected with clinical MAP UCF4 (1 × 10^7^ CFU/mL), followed by 24 h of incubation under the same conditions.

### 2.3. Measurement of IL-1β and TNF-α Expression in Cultured THP-1 Macrophages

RNA was isolated from each 2.0 mL sample of monocyte-derived macrophages following 24 h of infection with clinical MAP strain (UCF4). RNA was extracted using the RNeasy^®^ Mini Kit (Qiagen, Hilden, Germany) according to manufacturer protocols. RNA concentrations were measured using NanoDrop (OD at 260 nm). RNA was then reverse-transcribed to cDNA cDNA was synthesized from 1000 ng of each RNA sample using 5.8 µL master mix made from the High-Capacity cDNA Reverse Transcription Kit (Applied Biosystems, Waltham, MA, USA) and then topped up to a total volume of 20 µL with RNase-free water, according to manufacturer protocols. A thermal cycler (MyGene Series Peltier Thermal Cycler) was used to perform the reactions for 5 min at 25 °C, 20 min at 46 °C, and 1 min at 95 °C. The cDNA samples were stored at −20 °C or used immediately for RT-qPCR analysis. Gene expression was measured using specific primers for GAPDH, TNF-α, and IL1β, obtained from Bio-rad (Hercules, CA, USA), followed by quantitative reverse transcription PCR (RT-qPCR) analysis. For each sample, 5 µL of cDNA was mixed with 10 µL of PowerUp SYBR Green Master Mix (ThermoFisher Scientific, Waltham, MA, USA), 1 µL primer mix, and 4 µL of DEPC-treated water. Samples were added in triplicate to a 96-well microamp RT-PCR reaction plate, and the experiment was run using the 7500 Fast Real-Time PCR System (Applied Biosystems, Waltham, MA, USA). GAPDH was the control used to obtain baseline CT readings. Relative mRNA expression levels were calculated using the equation (2^−∆∆CT^).

### 2.4. Quantification of THP-1 Macrophage Viability and Apoptosis during MAP Infection

THP-1 macrophages were cultured in 100 µL media on a 96 well opaque-sided plate. All cultured cells used the specialty RPMI 1640 formulations described previously. Macrophages were administered 50 ng/mL PMA and kept in a humidified incubator at 37 °C and 5% CO_2_ for 48 h. Macrophages were infected following this incubation with clinical MAP at 1 × 10^7^ CFU/mL and maintained for 24 h. We then conducted one of two assays on the plated cells. First, we used the RealTime-Glo™ MT Cell Viability Assay (Promega, Madison, WI, USA), an ATP-based luminescence assay according to manufacturer protocols. Briefly, we mixed MT Cell Viability Substrate and NanoLuc^®^ Enzyme to 2X concentrations in folate and B_12_-free RPMI 1640 media and added 100 µL to each well. Cells were incubated with the reagent for 1 h, and then luminescence was measured using the GloMax Navigator system GM-2000 (Promega, Madison, WI., USA) Next, we used the RealTime-Glo™ Apoptosis Assay (Promega, Madison, WI, USA) an annexin-V-based luminescence assay. Briefly, we combined 2X concentrations of Annexin NanoBiT^®^ Substrate, CaCl_2_, Annexin V-SmBiT^®^, and Annexin V-LgBiT in prewarmed folate and B_12_-free RPMI 1640. 100 µL of the assay mixture were administered to each well and incubated for 20 min, and then the same instrument was used to measure luminescence. Both luminescence readings were analyzed to determine cell viability and apoptosis.

### 2.5. Culture and Treatment of Caco-2 Monolayers with Infected THP-1 Supernatant 

The impact of vitamin concentration on cell death and oxidative stress was examined in an immortalized enterocyte-like cell line (Caco-2 ATCC HTB-37.) Cells were cultured in ATCC-formulated Eagle’s Minimum Essential Medium (EMEM) supplemented with 20% FBS (ATCC, Manassas, VA, USA) and maintained at 37 °C in a humidified 5% CO_2_ incubator. 3 × 10^5^ cells were seeded in the base of clear-bottomed, opaque-sided 96-well plates with 200 μL media. The cells were allowed to differentiate for 14 days before the media was changed to the RPMI 1640 media with variable levels of folic acid and vitamin B_12_ (Table 1). On day 18, THP-1 macrophages were plated and differentiated for 48 h in RPMI 1640 media matching the vitamin concentrations of their paired Caco-2 monolayers. On day 20, the macrophages were infected with MAP as previously described. On day 21, supernatants were collected from each macrophage culture and centrifuged for 1 min at 8000 rcf to pellet debris and intact bacteria. Media was then removed from the Caco-2 wells and replaced with supernatant from infected macrophages with the same vitamin concentrations.

### 2.6. Quantification of Cytotoxicity and Oxidative Stress in Caco-2 Monolayers 

After preparation of the Caco-2 monolayers and administration of the infected THP-1 macrophage supernatant, we used the LDH-Glo™ Cytotoxicity Assay and the NADP/NADPH-Glo™ Assay (Promega, Madison, WI, USA) to quantify plasma membrane damage and oxidative stress, respectively. Briefly, for the former assay, all components were combined in warm media and administered to intact, adherent cells inside their respective wells. The cells were incubated for 30 min, and luminescence was measured with the GloMax Navigator™ (Promega, Madison, WI, USA) For the latter assay, cells were lysed with 0.2 N NaOH solution with 1% DTAB, and the lysate was separated to be subjected to heat under acidic or basic conditions to decompose NADPH or NADP^+^, respectively, according to manufacturer protocols. The heated lysate was then cooled to room temperature and administered the assay components. After a 30 min incubation, luminescence was again quantified with the GloMax™ Navigator. Cells were not reused between assays. All experimental groups were plated and assayed in triplicate.

## 3. Statistical Analysis

GraphPad Prism V.9.4.0 (GraphPad, La Jolla, CA, USA) was used for statistical analysis. First, the Kolmogorov–Smirnov normality test was used to assess normal distribution for all values. Following this analysis, we used Student’s *t*-test was to assess significance between two groups of values. One-way ANOVA was used to assess significance in studies with multiple experimental groups, followed by Sidak’s multiple comparisons test. All data are expressed as average ± SD of the mean, and the difference between treated samples vs. controls was considered statistically significant at *p*-value < 0.05 and 95% confidence interval. All experiments save ELISAs of plasma samples were performed in triplicates unless noted otherwise.

## 4. Results

### 4.1. Folate and Vitamin B_12_ Are Reduced in MAP-Positive CD Patients

**Figure 1:**
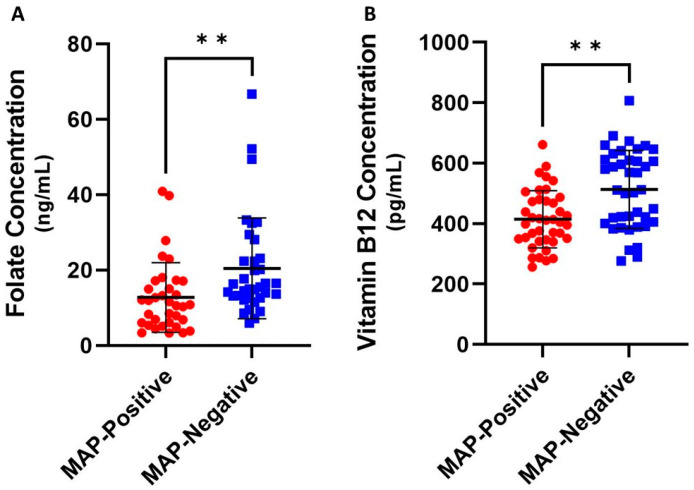
Levels of plasma folate (**A**) and B_12_ (**B**) in MAP-negative and MAP-positive CD patients, *N* = 35 for both groups. All measurements were performed in duplicates. ** Indicates *p*-value of less than 0.01.

While previous studies have established that CD patients are at an elevated risk of folate and B_12_ deficiency, there is a lack of data on how MAP-positive and MAP-negative CD patient subsets compare. As such, we took plasma samples from patients previously confirmed to be MAP-negative or MAP-positive via *IS900* PCR and compared their folate and B_12_ levels via ELISA. We found that average MAP-positive plasma folate levels were significantly reduced to 14.48 ± 13.88 ng/mL from MAP-positive 24.15 ± 25.74 ng/mL [Figure 1A] In addition, there was a significant decrease in average vitamin B_12_ to 414.48 ± 94.60 pg/mL from 512.86 ± 129.12 pg/mL in MAP-positive versus MAP-negative patients [Figure 1B]. Since the plasma samples were collected from two subsets of CD patients, these results indicate that the risk of malabsorptive folate and B_12_ deficiency may be correlated particularly with MAP infection in CD patients.

### 4.2. THP-1 Macrophages Cultured in Folate and B_12_ Supplemented and Deficient Media Show Altered Cytokine Expression

**Figure 2:**
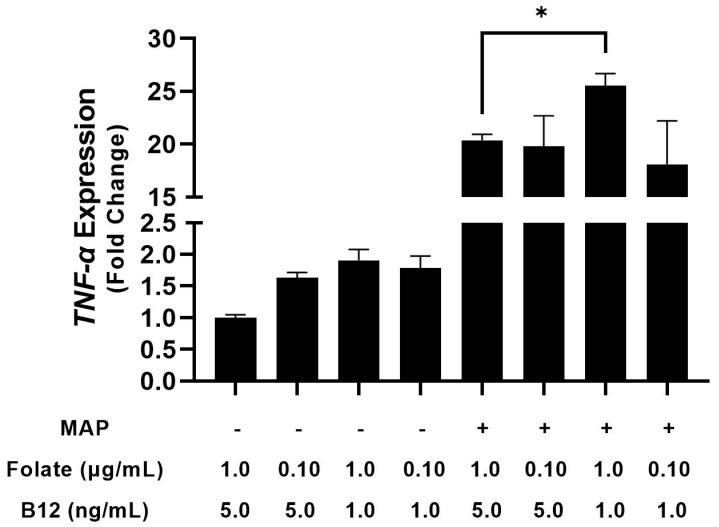
Effect of folate and B_12_ deficiency on TNF-α expression in MAP-infected and uninfected macrophages. * Indicates *p*-value of less than 0.05.

**Figure 3:**
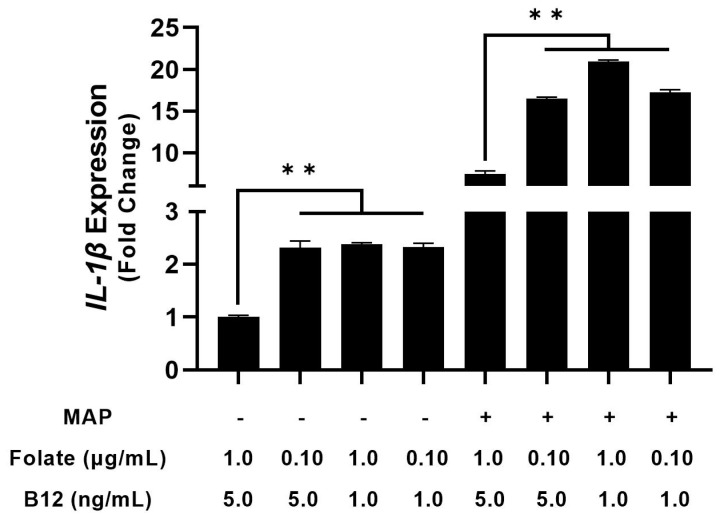
Effect of folate and B_12_ deficiency on IL-1β expression in MAP-infected and uninfected macrophages. ** Indicates *p*-value of less than 0.01.

Next, we considered how differing vitamin levels in cell culture media alter macrophage cytokine expression. To examine this phenomenon, we took folate and B_12_-free RPMI 1640 and manually supplemented it with folate and B_12_ to yield folate-low media, folate-high media, B_12_-low media, B_12_-high media, folate/B_12_-low media, folate/B_12_-high media, and ordinary RPMI 1640. After 48 h of PMA stimulation and differentiation in the depleted media, we infected them with clinical MAP for 24 h and examined relative cytokine mRNA. folate, B_12_, and folate/B_12_ deficiency increased TNF-α expression in uninfected macrophages by 1.63 ± 0.08-fold, 1.91 ± 0.17-fold, and 1.79 ± 0.19-fold, respectively; however, these effects were not statistically significant [Figure 2]. In infected macrophages, B_12_-deficiency increased TNF-α expression from 20.37 ± 0.54-fold to 25.52 ± 1.17-fold [Figure 2]. However, no other vitamin deficiency, even concurrent folate/B12, significantly altered TNF-α expression. By contrast, folate, B12, and folate/B_12_ deficiency all had inhibitory effects on IL-1β expression in uninfected (2.31 ± 0.13, 2.38 ± 0.03, and 2.32 ± 0.07-fold, respectively) and infected macrophages (16.49 ± 0.16, 20.93 ± 0.17, and 17.26 ± 0.30-fold, respectively, compared with untreated 7.41 ± 0.38) [Figure 3].

**Figure 4:**
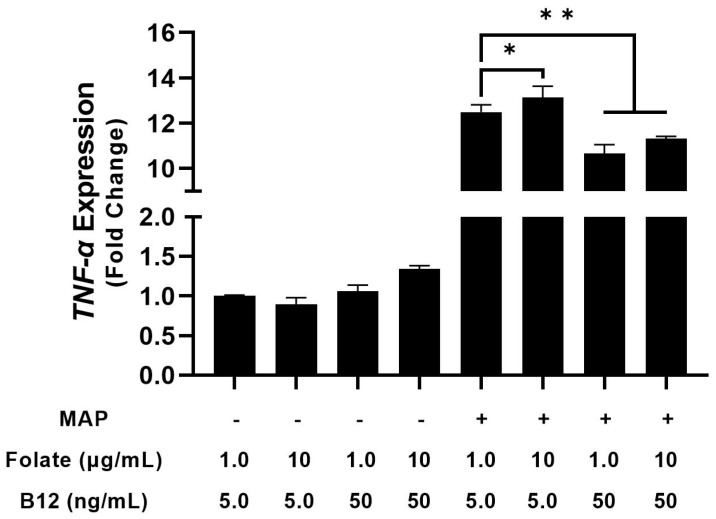
Effect of folate and B_12_ supplementation on TNF-α expression in MAP-infected and uninfected macrophages. * Indicates *p*-value of less than 0.05. ** Indicates *p*-value of less than 0.01.

**Figure 5:**
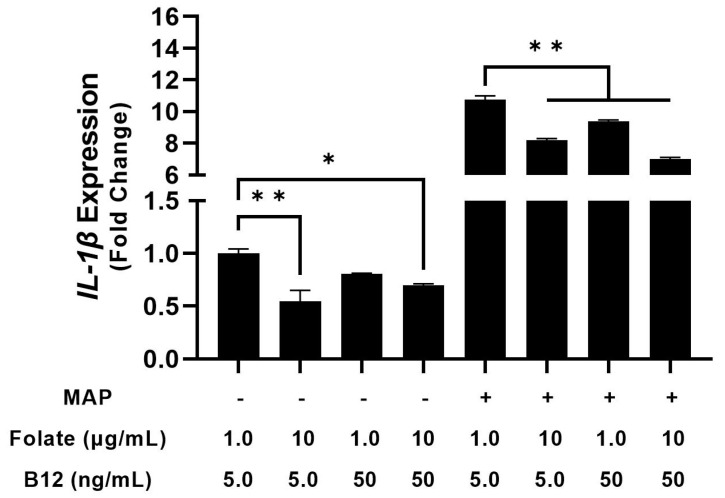
Effect of folate and B_12_ supplementation on IL-1β expression in MAP-infected and uninfected macrophages. * Indicates *p*-value of less than 0.05. ** Indicates a *p*-value of less than 0.01.

We followed these experiments with another analysis to examine supplementation with folate and B12. Tenfold elevation of B_12_ in culture medium for infected macrophages reduced TNF-α expression from 12.49 ± 0.33 to 10.66 ± 0.39-fold, while folate had no significant effect (13.14 ± 0.50) [Figure 4]. Folate and B_12_ supplementation alone had no significant effect on TNF-α expression in uninfected macrophages (with a 0.89 ± 0.09 and a 1.06 ± 0.07-fold change, respectively) [Figure 4]. Interestingly, concurrent folate/B_12_ supplementation increased TNF-α expression by 1.34 ± 0.04-fold in uninfected macrophages but reduced it from 12.49 ± 0.33 to 11.31 ± 0.10 in infected macrophages [Figure 5]. Elevated folate reduced IL-1β expression from 1.00 ± 0.01 to 0.55 ± 0.10-fold in uninfected macrophages and from 10.75 ± 0.25 to 8.19 ± 0.11-fold in infected macrophages [Figure 5] Supplementation with B_12_ reduced IL-1β expression from 1.00 ± 0.04 to 0.80 ± 0.01-fold in uninfected macrophages and from 10.75 ± 0.25 to 9.39 ± 0.09-fold in infected macrophages [Figure 5]. Concurrent folate/B_12_ supplementation’s effect is more uniform in IL-1β than TNF-α, which shows a reduction from 1.00 ± 0.04 to 0.69 ± 0.02-fold in uninfected macrophages and from 10.75 ± 0.25 to 7.00 ± 0.10-fold in infected macrophages [Figure 5]. These results indicate that folate and B_12_ supplementation have an anti-inflammatory effect on macrophage cytokine expression during MAP infection, with results varying by specific cytokine. By contrast, folate and B_12_ deprivation enhance the inflammatory response in identically treated cells.

### 4.3. MAP Infection Increases Apoptosis during Folate and B_12_ Supplementation and Decreases Apoptosis during Depletion

**Figure 6:**
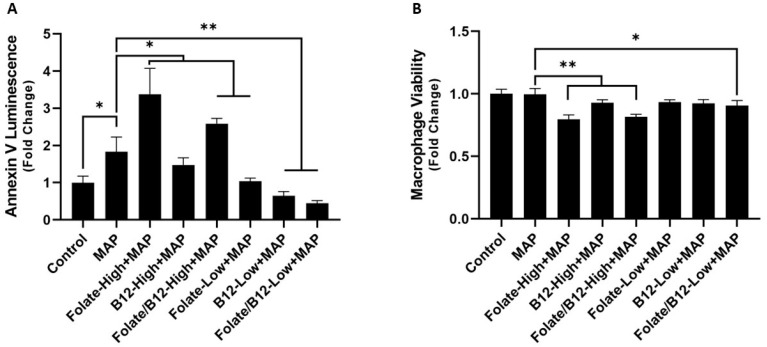
Effect of folate and B_12_ supplementation or depletion on macrophage apoptosis (**A**) and general viability (**B**) during MAP infection. * Indicates *p*-value of less than 0.05. ** Indicates *p*-value of less than 0.01.

Apoptosis of infected cells is a critical immune mechanism for countering intracellular pathogens, and MAP consequently uses a suite of anti-apoptotic proteins to impede it [44]. Accordingly, we hypothesized that infected macrophages supplemented with folate and B_12_ would increase apoptosis to control the infection. We used two methods to assess the apoptosis of MAP-infected macrophages. First, we directly quantified apoptosis using annexin V luminescence. MAP infection increased apoptosis by 1.83 ± 0.40 fold compared to uninfected controls, as anticipated. Furthermore, folate-enriched media increased macrophage apoptosis during infection by 3.38 ± 0.07-fold, a significant increase compared to MAP infection in control media [Figure 6A]. An increase in apoptosis to 2.58 ± 0.14-fold was also observed in concurrent folate and B_12_ supplemented media [Figure 6A]. We did not observe a significant change in media supplemented with B_12_ alone. Depletion of folate, B12, and both folate and B_12_ had the inverse effect: annexin V luminescence decreased to 1.04 ± 0.08, 0.64 ± 0.12, and 0.45 ± 0.07-fold, respectively [Figure 6A]. 

We followed this experiment with an ATP-based viability assay. General macrophage viability after MAP infection declined to 0.80 ± 0.05 and 0.82 ± 0.02-fold in folate and concurrent folate/B_12_ supplemented media, corresponding with the observed increases in annexin V luminescence [Figure 6B]. Interestingly, there was no significant change in macrophage viability in the MAP-infected cells compared with the uninfected cells. Similarly, decreased annexin V in folate, B_12_, and folate/B_12_ deficient media did not correspond with a change in overall viability in these groups [Figure 6B]. However, there was a small but statistically significant decrease in overall macrophage viability in the folate/B_12_-low group, from 1.00 ± 0.05-fold to 0.91 ± 0.04-fold.

### 4.4. Folate and Vitamin B_12_ Deficiency Individually Exacerbate Cytotoxicity in Caco-2 Monolayers

After assessing the effects of folate and vitamin deficiency on macrophage inflammation, we considered the impact of altered macrophage inflammation on enterocytes. We hypothesized that changes in macrophage survival and pro-inflammatory cytokine expression would have a detrimental impact on co-cultured Caco-2 cells. Accordingly, we differentiated Caco-2 cells into monolayers and maintained them in culture for 7 days in RPMI 1640 with altered vitamin levels. administered supernatant collected from macrophages cultured and infected in media with comparably altered vitamin levels. 24 h post-treatment, we examined LDH release and NADP^+^/Total NADP in the monolayers.

**Figure 7:**
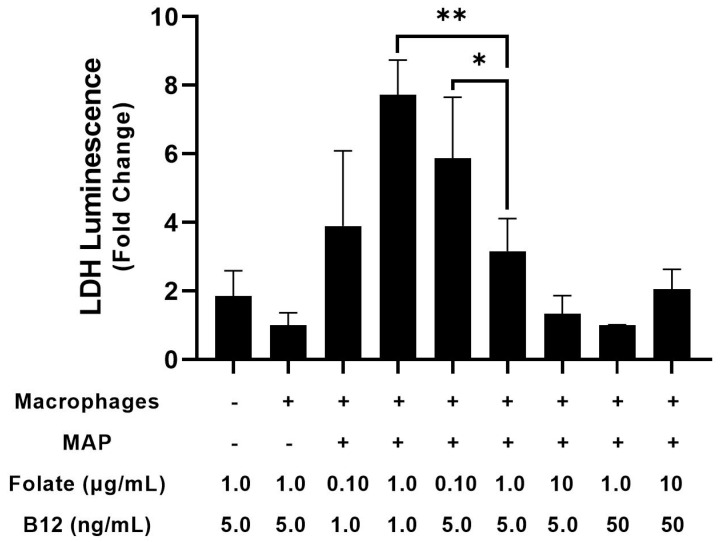
Effect of folate and vitamin B_12_ concentration on LDH release from Caco-2 monolayers after treatment with supernatant from infected macrophages. * Indicates *p*-value less than 0.05. ** Indicates *p*-value less than 0.01.

Treating Caco-2 cells with supernatant from infected macrophages increased LDH luminescence 3.16 ± 0.95-fold compared with uninfected controls, though this effect did not reach statistical significance [Figure 7]. By contrast, administration of infected macrophage supernatant in vitamin B_12_-low conditions increased LDH luminescence 7.73 ± 1.01-fold, which was significant compared to both infected and uninfected cells in control media [Figure 7]. Similarly, folate-low media increased luminescence by 5.87 ± 1.78-fold compared with the control [Figure 7]. Interestingly, concurrent folate and vitamin B_12_ deprivation did not significantly alter LDH luminescence in culture. Treating Caco-2 cells with supernatant from infected macrophages did not significantly alter the percentage of NADPH compared with the control in any group, suggesting that oxidative stress did not mediate the damage observed in the LDH assay [Table 2].

## 5. Discussion

CD patients are at an elevated risk of malabsorptive folate and vitamin B_12_ deficiency [21,32]. Furthermore, attempts to avoid foods that patients believe to be triggers for CD relapse can lead to self-imposed dietary restriction and reduced nutrient intake [45]. Accordingly, periodic screenings for these vitamins are indicated for CD patients, particularly those who received ileal resection [40]. However, there is a dearth of information on the effects of folate and vitamin B_12_ supplementation on CD symptoms and inflammation. Furthermore, the state of the literature is murky on the effects of folate and B_12_ supplementation on common CD pathogens like MAP. Physicians with CD patients are left without guidance about the risks of prolonged folate and B_12_ deficiency in this patient subset and a potential tool to mitigate inflammation.

Gastrointestinal health and resilience against infection have been correlated with folate and vitamin B_12_ intake. Rat models of methyl donor deficiency indicate that methyl donor-low diets impede bowel development and barrier function while aggravating induced colitis [18,46]. By contrast, folate-producing lactic acid bacteria have an anti-inflammatory effect on induced murine mucositis [47]. Additionally, murine diets supplemented with folate and other methyl-donating nutrients improved antimicrobial gene expression and resilience against adherent invasive *E. coli* in a mouse model of CD [36]. These findings concur with a study establishing that neonatal folate deprivation sensitizes adult guinea pigs to Shigella infection [48]. In humans, folate-associated metabolic pathways are perturbed in pediatric CD patients, and a review of meta-analyses for CD environmental risk factors indicated that high folate levels were protective [49,50]. Furthermore, low folate levels in CD were associated with increased CD activity in a Swiss cohort of patients [26].

The results of our investigation add to these findings by showing that folate and B_12_ alter inflammation during infection with a common CD pathogen. MAP-positive CD patients have significantly lower plasma folate and B_12_ than MAP-negative CD patients. We further show that pro-inflammatory cytokines IL-1β and TNF-α are significantly upregulated during folate and vitamin B_12_ deprivation in after MAP infection, while supplementation significantly reduces their expression. Macrophages in folate and B_12_-deficient media are less likely to undergo apoptosis during MAP infection, suggesting this key mechanism to counteract intracellular pathogens is inhibited during vitamin deficiency. Folate supplementation, by contrast, increased apoptosis of infected macrophages. Finally, the treatment of enterocytes with folate-low or B_12_-low supernatant from infected macrophages leads to increased LDH release without significant alterations in oxidative stress.

Our findings on folate and B_12_ in CD patient plasma cannot at this time be compared with normal clinical serum ranges for these vitamins, as the ELISAs used to quantify folate and vitamin B_12_ availability are not approved for clinical practice. However, these results indicate that even within a patient group at risk for folate and B_12_ deficiency, distinct subsets of folate and B_12_ availability can be characterized based on MAP infection. Furthermore, the data on cytokine availability indicate that folate and B_12_ levels affect macrophage-mediated inflammation after only 48 of h prophylactic exposure. Since monocyte-derived macrophages constitutively infiltrate the gut, these results suggest that clinical trials of folate and B_12_ supplementation may gradually impact inflammation patterns as older, vitamin-restricted cells are replaced [51]. Importantly, our work builds on the findings of Samblas and colleagues by showing that the anti-inflammatory effects of folate and B_12_ can be observed during an ongoing bacterial infection, not just after stimulation with a TLR agonist like LPS [30]. Since these findings are paired with improved apoptosis of infected macrophages, we propose that MAP-infected CD patients taking folate and B_12_ supplements may experience reduced inflammation upon the initial macrophage encounter with MAP. Altered inflammation may be followed by apoptosis of the pro-inflammatory, MAP-infected cells, resulting in the removal of both pathogen and inflammatory mediator.

Future studies are required to explore not merely how folate and B_12_ alter MAP infection in cell culture, but in animal and clinical studies. Our study centered on monocyte derived macrophages because prior studies have highlighted macrophages as a crucial innate immune mediator for both MAP destruction and proliferation [52]. However, we do not analyze other phagocytic immune cells or determine if the 48 h differentiation period is the only relevant window to mediate folate and B_12_’s anti-inflammatory effects. Furthermore, macrophages frequently interact with components of the adaptive immune system, thereby alter adaptive immunity. We do not explore how folate and B_12_ might affect this function to contribute to long-term inflammatory CD patterns. Accordingly, further investigation is warranted.

## Figures and Tables

**Table 1:** Concentrations of folate and B_12_ in modified RPMI 1640 media for macrophage cell culture and infection.

Culture Condition	Folate (μg/mL)	B_12_ (ng/mL)
Control RPMI 1640	1.0	5.0
Folate-High	10.0	5.0
B_12_-High	1.0	50.0
Folate + B_12_-High	10.0	50.0
Folate-Low	0.10	5.0
B_12_-Low	1.0	1.0
Folate + B_12_-Low	0.10	1.0

**Table 2:** Effect of folate and vitamin B_12_ concentration on NADP release from Caco-2 monolayers after treatment with supernatant from infected macrophages.

Infection and Treatment	NADP^+^/(NADPH + NADP^+^) × 100 ± SD
Control (no infection)	6.23 ± 0.396
MAP infection (1 × 10^7^ CFU/mL)	6.89 ± 0.761
Folate-low + MAP infection	7.74 ± 0.750
B_12_-low + MAP infection	8.24 ± 0.657
Folate/B_12_-low + MAP infection	7.07 ± 0.801
Folate-high + MAP infection	7.63 ± 1.04
B_12_-high + MAP infection	6.59 ± 1.00
Folate/B_12_-high + MAP	6.80 ± 0.716

## Data Availability

The original contributions presented in this study are included in the article. Further inquiries may be directed to the corresponding author.
